# Whole-exome sequencing of a novel initiation codon mutation in *RUNX2* in a Chinese family with cleidocranial dysplasia

**DOI:** 10.1097/MD.0000000000027746

**Published:** 2021-11-12

**Authors:** Liyuan Yang, Genqi Lu, Wenjing Shen, Wenjing Chen, Haiyan Lu, Guozhong Zhang, Shuo Yuan, Shushen Zheng, Jiabao Ren

**Affiliations:** a Department of Prosthodontics, Hebei Key Laboratory of Stomatology, Hebei Clinical Research Center for Oral Diseases, School and Hospital of Stomatology, Hebei Medical University, Shijiazhuang, PR China; b Department of Orthodontics, Hebei Key Laboratory of Stomatology, Hebei Clinical Research Center for Oral Diseases, School and Hospital of Stomatology, Hebei Medical University, Shijiazhuang, PR China; c College of Forensic Medicine, Hebei Medical University, Shijiazhuang, PR China; d Xingtai Medical College, Xingtai, PR China.

**Keywords:** cleidocranial dysplasia, initiation codon variant, *RUNX2* gene, whole-exome sequencing

## Abstract

Cleidocranial dysplasia (CCD) is mainly attributable to a variant of runt-related transcription factor 2 (*RUNX2*) on chromosome 6p21. CCD is an autosomal dominant skeletal disorder characterized by open/delayed closure of fontanels, clavicular hypoplasia, retention of deciduous teeth, and supernumerary permanent teeth. The aim of this study was to investigate potentially pathogenic mutations in 2 Chinese families. Genomic DNA was obtained from peripheral blood lymphocytes, and whole exome sequencing and Sanger sequencing were performed to detect gene variants. Real-time quantitative PCR was performed to determine the mRNA expression level of *RUNX2* in the proband of family 1. Silico algorithms and conservation analyses were used to evaluate the functional impact. We identified a novel initiation codon mutation (c.2T>C) and a previously reported mutation (c.569G>A). Familial co-segregation verified an autosomal-dominant inheritance pattern. Our findings demonstrated that the novel mutation c.2T>C causes CCD. Quantitative real-time PCR suggested that downregulated RUNX2 levels and haploinsufficiency in RUNX2 lead to CCD. These results extend the spectrum of RUNX2 mutations in CCD patients and can be used for genetic consultation and prenatal diagnosis.

## 1 Introduction

Cleidocranial dysplasia (CCD, MIM 119600), with a prevalence rate of 1:1,000,000, is a rare autosomal dominant skeletal dysplasia characterized by defective skull ossification, open/delayed closure of fontanels, clavicular hypoplasia, delayed ossification of the pelvis, short stature, retention of deciduous teeth, and supernumerary permanent teeth.^[1–4]^ The phenotypes of CCD have wide variations, ranging from patients with only mild dental deformities to those with severe bone abnormalities.^[3,4]^ Environmental and epigenetic factors may contribute to the diverse phenotypes.^[5]^

Variants in the runt-related transcription factor 2 gene (*RUNX2*, MIM 600211) account for 60% of the CCD cases.^[6,7]^*RUNX2* is located on chromosome 6p21 and has 8 coding exons.^[6]^ RUNX2 is a key regulator of osteoblast differentiation and bone development.^[7]^ Homozygous Runx2 knockout mice (Runx2^−/−^) exhibited a lack of osteoblasts, failed in both intramembranous and endochondral ossification, and showed early lethality after birth. In contrast, heterozygous Runx2 knockout mice (Runx2^+/−^) displayed a phenotype similar to that of CCD patients with respect to hypoplastic clavicles and defective skull formation.^[8,9]^

Currently, the Human Gene Mutation Database (HGMD Professional 2020.4 http://www.hgmd.cf.ac.uk/) has 202 registered *RUNX2* variants responsible for CCD with various severities, namely, missense/nonsense (80), splicing (11), small/gross deletions (69), small insertions (28), gross insertions/duplications (8), indels (2), and complex rearrangements (4). However, no apparent genotype–phenotype correlations have been investigated, and only a few RUNX2 mutations have been reported in Chinese CCD patients.

In the present study, we have reported a novel initiation codon variant in 1 *RUNX2* allele (c.2T>C) as well as a previously reported missense mutation in 2 Chinese Han families with CCD.

## 2 Material and methods

### 2.1 Ethical approval and subjects

**Figure 1:**
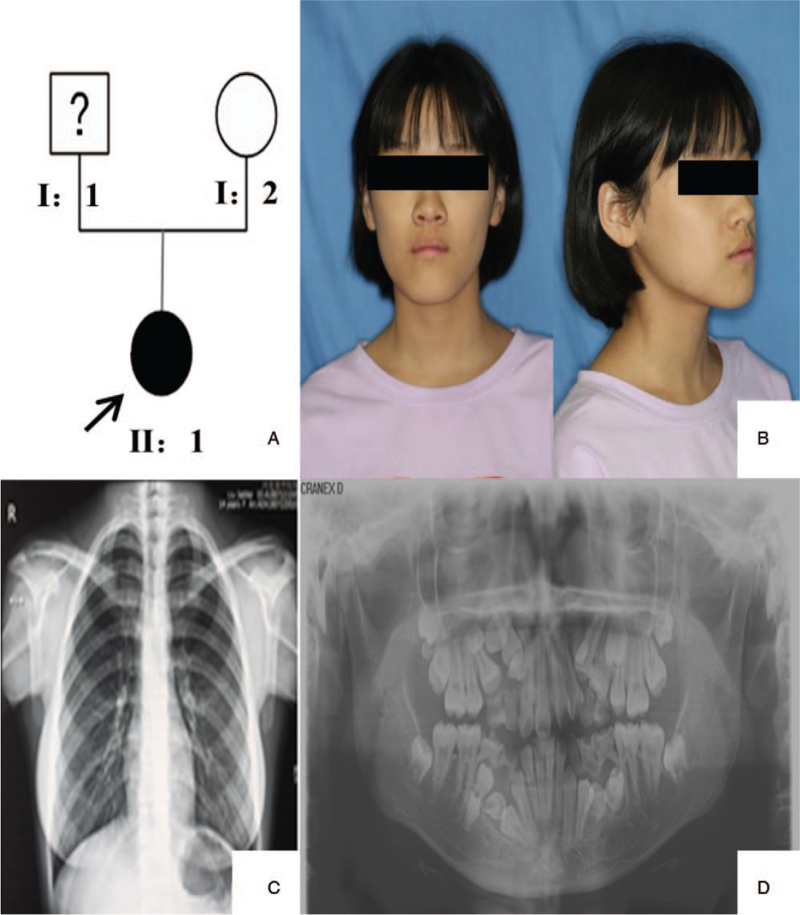
Examination results for the proband in family 1. (A) Pedigree of the family of the proband. The arrow shows the proband: patient II:1, a 15-year-old girl. (B) Facial photographs of the proband. (C) Bilateral aplasia of the clavicles observed in the chest radiograph of the proband. (D) Retained deciduous teeth and delayed eruption of permanent teeth detected in the panoramic radiograph of the proband.

**Figure 2:**
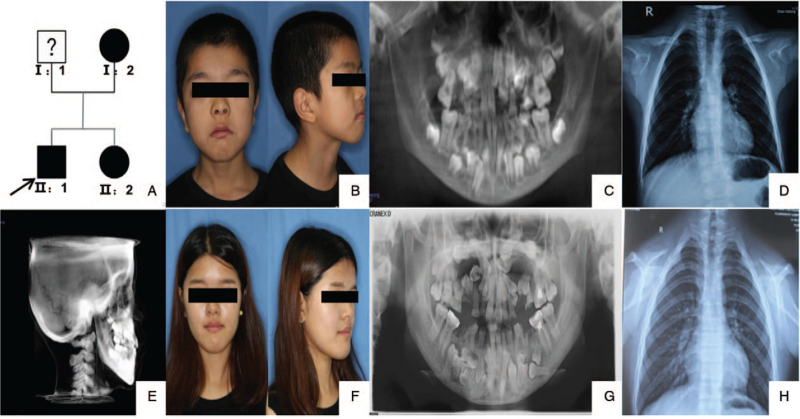
Examination results for the patients in family 2. (A) Pedigree of family 2. The arrow shows the proband. (B) Patient II:1 (proband): a 10-year-old boy in family (C) Retained deciduous teeth and delayed eruption of permanent teeth detected in the panoramic radiograph of the proband. (D) Bilateral hypoplasia of the clavicles and scoliosis found in the chest radiograph of the proband. (E) The frontal fontanelle of the proband has a delayed or open skull suture, and the middle part of the face is not fully developed. (F) Facial photographs of patient II:2: the elder sister of the proband. (G) Retained deciduous teeth and delayed eruption of permanent teeth detected in the panoramic radiograph of patient II:2. (H) Normal development with bilateral locking and spine found in the chest radiograph of patient II:2.

This work was approved by the Ethics Committee of the Affiliated of Stomatology Hospital of Hebei Medical University (No: [2016]004). All participants or their guardians signed written informed consent. The probands (Fig. 1, Family 1, II-1; Fig. 2, Family 2, II-1) were initially evaluated and diagnosed with CCD by Hebei Children's Hospital, and they came to the Hospital of Stomatology, Hebei Medical University, for dental treatment. All family members were checked by 2 experienced dentists, who performed oral and radiographic examinations.

### 2.2 DNA sample collection and extraction

The QIAmp DNA Blood Midi kit (Cat#51185; Qiagen, Hilden, Germany) was used to obtain genomic DNA from the peripheral blood lymphocytes of all participants, as per the manufacturer's protocols. DNA purity was analyzed with the NanoPhotometer spectrophotometer (Implen, Westlake Village, CA) and quantified with the Qubit DNA assay kit and Qubit 2.0 fluorimeter (Life Technologies, Carlsbad, CA).

### 2.3 Whole exome sequencing and Sanger sequencing

Whole exome sequencing was performed for the probands. Target enrichment and amplification were performed using the liquid-phase capture method with testing kits from iGeneTech (Beijing, China). The NovaSeq 6000 platform (Illumina Inc, Santiago, CA) was used to sequence exons from the targeted regions. With a sequencing yield of more than 14,555.7 Mb raw bases, the samples achieved a mean target depth of 140×. The reads were aligned to Genome Reference Consortium Human Build 37 (GRCh37/hg19) with Burrows-Wheeler Aligner, and single-nucleotide variants and small insertions and deletions (indels) were identified with SAMtools (http://www.htslib.org/). Single-nucleotide polymorphisms and indels were identified using the Genome Analysis Toolkit and annotated with ANNOVAR (https://annovar.openbioinformatics.org/). Candidate variants were filtered according to the following criteria: MAF<1% and exonic. The candidate mutation of *RUNX2* was verified with Sanger sequencing. Primers of exon2 of *RUNX2* (reference sequence NM_022336.4) were as follows: F: 5′-GTCACTACCAGCCACCGAGA-3′; R: 5′-TTGGAAAAGCTAGCAGTTTATCA-3′. Primers of exon4 were: F: 5′-AGTGGCATCACAACCCATACA-3′; R: 5′-CACTCAACTTCATCTGGATGTATTT-3′. The reference sequence for *RUNX2* was NM_001024630.4. We identified the nucleotide variant in *RUNX2*, and we used 100 unrelated population-matched controls.

### 2.4 Quantitative real-time PCR for mRNA expression of RUNX2

The cDNAs were produced; Maxima SYBR Green/ROX qPCR Mastermix (Cat#K0221; Thermo Fisher Scientific) and ABI7500 real-time PCR system (Applied Biosystems) were used to check relative mRNA expression levels, which were evaluated with the 2–ΔΔCt method. Glyceraldehyde 3-phosphate dehydrogenase (GAPDH) was used as the internal control. The following primers were used for qPCR: *RUNX2* forward, 5′-AAGTAGCAAGGTTCAACGATCTG-3′, and reverse, 5′-TTCCCGAGGTCCATCTACTG-3′; GAPDH forward, 5′-TGGGTGTGAACCATGAGAAGT-3′, and reverse, 5′-TGAGTCCTTCCACGATACCAA-3′.

### 2.5 Bioinformatics analyses

Pathogenicity of the novel variant was predicted using Sorting Intolerant from Tolerant (SIFT, http://sift.jcvi.org), polymorphism phenotyping (PolyPhen-2, http://genetics.bwh.harvard.edu/pph2/), and MutationTaster (http://www.mutationtaster.org). Conservation analysis of the RUNX2 missense variants in different species was performed using Clustal Omega (https://www.ebi.ac.uk/Tools/msa/clustalo) with reference to the following species: human (>NP_001015051.3), rhesus monkey (>XP_028703335.1), chimpanzee (>XP_016811118.1), chicken (>XP_025004234.2), rat (>NP_001265412.1), and mouse (>NP_001139510.1).

## 3 Results

### 3.1 Clinical manifestations

The clinical features of the patients and phenotypic characteristics of the probands were as follows: short stature, broad forehead, frontal bossing, orbital hypertelorism, midface hypoplasia, and protruding mandible (Fig. 1B, Fig. 2B & F). Failure of eruption of permanent teeth, retention of deciduous teeth, and supernumerary teeth were confirmed by panoramic radiograph. Tooth deformity was observed because of the failure of eruption of several supernumerary teeth in the mandible or maxilla (Fig. 1D; Fig. 2C & G). Aplasia of the clavicle (Fig. 1C; Fig. 2D & H) and delayed closure of cranial sutures in II:1 proband of family 2 were detected in the chest radiograph (Fig. 2E). On the basis of the clinical and radiological results, CCD was confirmed, and the clinical results of the other affected family members are listed in Table 1.

**Table 1:** Clinical and genetic features of the patients with cleidocranial dysplasia.

	Family 1	Family 2	
	II:1	II:1	II:2
Clinical features			
Age	15	10	20
Sex	F	M	F
Height (cm)	162	140	166
Family history	+	+	+
Short stature	−	+	−
Delayed or open skull suture	−	+	+
Wormian bone	+	+	+
Frontal bossing	−	−	−
Hypertelorism	+	+	+
Hypoplasia of the maxilla	+	+	+
Clavicle hypoplasia	+	+	+
Wide pubic symphysis			
Short middle phalanx of the 5th finger	−	+	−
Vertebral alteration (scoliosis)	−	+	−
Delayed eruption of permanent teeth	+	+	+
Impacted tooth	+	+	+
Malocclusion	+	+	+
Genetic features			
Codon	M2T	R190Q	R190Q
Mutation type	Nonsense	Missense	Missense
Nucleotide change	c.2T>C	c.569G>A	c.569G>A
Location	AD1	Runt	Runt

+: presence; −: absence.

### 3.2 Analysis of RUNX2 variants

**Figure 3:**
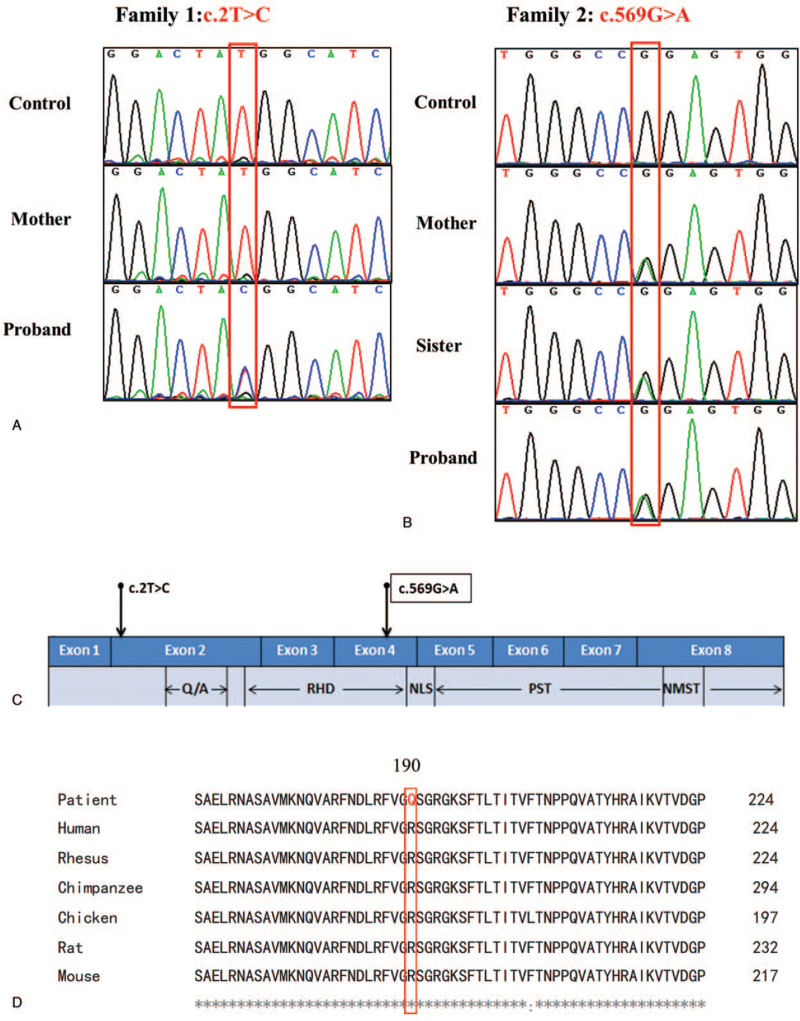
Sequencing results show RUNX2 mutations in the 2 families. (A) A novel transition from thymine to cytosine at the second base of the initiation codon (c.2T>C) in *RUNX2* in family 1. (B) A previously reported mutation (c.569G>A, p.R190Q) in the proband, his elder sister, and their mother. (C) Schematic representation of RUNX2 structure and annotated mutations. (D) The sequence alignment results show that the residues (c.569G>A) in RUNX2 is conserved across 6 species: the mutant allele is boxed.

We identified a novel heterozygous *RUNX2* missense variant c.2T>C (NM_001015051.4) that alters the initiation codon ATG to ACG in family 1 (Fig. 3A) and a previously reported mutation (c.569G>A, p.Arg190Pro) in family 2 (Fig. 3B). In family 1, the same mutation site was not detected in the patient's mother (Fig. 3A). We could not contact the patient's father because the parents were divorced, so the patient may have inherited the mutation from her father. Furthermore, the nucleotide alteration c.2T>C was not found in the healthy controls (n = 100) or NHLBI exome sequencing project Exome Variant Server (https://evs.gs.washington.edu/EVS/), indicating the substitution is a rare variant. Then, the candidate variant was confirmed for proband 1 and her mother by Sanger sequencing. In family 2, we found a previously reported mutation (c.569G>A, p.Arg190Pro)^[25]^ in the proband, his older sister, and their mother (Fig. 3B). Figure 3C shows the schematic representation of *RUNX2* structure and annotated variants. The mother was heterozygotic for *RUNX2*. We could not get in touch with the father. On the basis of the autosomal dominant inheritance pattern of CCD, the siblings’ mutation was inherited from their mother. The mutation (c. 569G>A) was shared by all affected members^[25]^ but not detected in 100 unrelated healthy Chinese volunteers.

### 3.3 RUNX2 mRNA expression

qPCR was performed to evaluate the variant effects and determine mRNA expression levels of *RUNX2* of the proband in family 1. GAPDH was used as the internal reference (control group was set to 1), and the relative quantity of *RUNX2* mRNA in the proband was 0.616. The results indicated that the mRNA levels of *RUNX2* were downregulated in the patient.

### 3.4 Bioinformatics analyses

SIFT, Polyphen2, and MutationTaster predicted that the mutation (c.2T>C) was “deleterious” (0.00), “possibly damaging” (0.838), and “disease-causing” (1.00), respectively, suggesting the variant is highly pathogenic. No other candidate variants were identified in the evaluation of the exome file of family 1 to rule out the possibility of the contribution of any variation in other known causative genes for CCD. A cross-species amino acid sequence alignment of the reported mutation (c.569G>A, [p.Arg190Pro]) showed that Arg190 was highly conserved among humans, rhesus macaques, chickens, mice, rats, and chimpanzees (Fig. 3D).

## 4 Discussion

CCD is a skeletal dysplasia that ranges from classical CCD (dental abnormalities, hypoplastic/aplastic clavicles, and delayed closure of cranial sutures) to mild CCD to isolated dental anomalies without the skeletal features.^[10]^ Among the 202 registered *RUNX2* variants (HGMD Professional 2020.4 http://www.hgmd.cf.ac.uk/), 99% (201/202) cause classical CCD. In this study, the 2 probands and family 2 members all showed classic features such as short stature, midface hypoplasia, frontal bossing, retention of deciduous teeth, failure of eruption of permanent teeth, aplasia of the clavicle, and supernumerary teeth, and the proband II:1 of family 2 exhibited delayed closure of cranial sutures. These phenotypes are consistent with previous findings in which most cases exhibited classical features.

In this study, we found a previously reported missense variant (p.Arg190Pro) in *RUNX2* that has been previously found to be responsible for CCD,^[11]^ but the mutation detected in the initiation codon (c.2T>C) has not yet been reported. The computational programs all predicted that the c.2T>C missense change is damaging to the resultant protein function and structure. Further, according to the criteria for classifying pathogenic variants proposed by ACMG,^[12]^ the initiation codon variant should be considered as very strong evidence of pathogenicity (PVS1).

**Figure 4:**
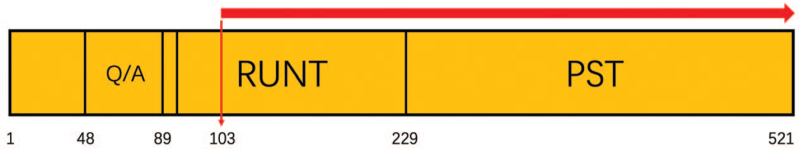
Prediction of the truncated protein of RUNX2 (c.2T>C). We hypothesized that the translation is mostly initiated 304 nucleotides downstream.

On the basis of Kozak principles,^[13]^ we hypothesized that the translation is mostly initiated 304 nucleotides downstream for type 2 RUNX2 (NM_001015051.3; Fig. 4). Consequently, this initiation codon mutation results in a frameshift mutation and truncated proteins (lack of the Q/A domain and part of the runt domain), which is consistent with the previous consensus that most mutations that cause premature termination in the runt domain produce a classic CCD phenotype.^[14]^ It is also possible that the mRNAs are degraded via the nonsense-mediated mRNA decay quality-control mechanism.^[15]^

The etiology of CCD is heterozygous variants in *RUNX2*, which encodes a transcription factor essential for osteoblast differentiation. Previous studies have shown that bones are malformed in case of a homozygous deletion in this gene in animals. Thus, the knockout mice with Runx2^−/−^ lack osteoblasts and bones,^[8]^ whereas the heterozygous mice (Runx2^+/−^) show abnormalities similar to those of CCD.^[9]^ Quantitative real-time PCR results showed that the *RUNX2* level was downregulated to 60% in patient II:1 family 1. Previous studies have shown a critical gene dosage requirement of functional RUNX2 for the formation of intramembranous bone tissues during embryogenesis. A decrease to 70% RUNX2 levels indicates CCD; in contrast, >79% levels produce a normal skeleton.^[14,16]^ Our result is consistent with those of previous studies^[6,16]^ and confirms the mechanism underlying this case is haploinsufficiency.

Of the previously reported 202 *RUNX2* variants, 63% (51/80) of the missense/nonsense mutations occurred in the runt domain, which is the most important variation hotspot of *RUNX2*.^[2,6,7,17–24]^ In this study, we located the variant (p.Arg190Pro) in the runt domain, confirming the above-mentioned point. Sequence alignment results showed that ARG190 is highly conserved. A functional study showed that the R190Q variant exhibited no DNA binding and markedly reduced transactivation activities.^[25]^ This genotype is correlated with the classic CCD phenotype^[25,26]^ and similar dental abnormalities.^[11,27]^ Interestingly, heterozygotic *Runx2* mice have no dental phenotypes, in contrast to the hyperdontia phenotype in humans; this may be attributable to composition differences in the mouse monophydont dentition, as it parallels the more simply patterned human primary dentition. A previous study demonstrated that *RUNX2* suppresses the expression of Wnt inhibitors in the dental mesenchyme; increased mesenchymal Wnt signaling inhibits the sequential formation of teeth and is attributable to supernumerary teeth caused by *RUNX2* variants in humans.^[6,16]^ However, the detailed mechanism underlying this process needs to be studied further.

## 5 Conclusion

This study demonstrated that a novel heterozygous initiation codon variant (c.2T>C) in *RUNX2* causes CCD. This study expands the pathogenic variant spectrum of *RUNX2* and could help in genetic counseling and prenatal screening and contribute to disease status prediction for CCD families.

## Acknowledgments

We sincerely thank all the subjects and medical staff involved in this study for their help in sample collection, diagnosis, and analysis. We are also very grateful to the College of Forensic Medicine of Hebei Medical University and Hebei Key Laboratory of Forensic Medicine for providing lab instruments and technical support.

## Author contributions

**Conceptualization:** Jiabao Ren, Wenjing Shen.

**Data curation:** Jiabao Ren, Wenjing Shen.

**Formal analysis:** Shuo Yuan.

**Funding acquisition:** Wenjing Shen.

**Investigation:** Wenjing Chen, Haiyan Lu, Shuo Yuan, Shushen Zheng.

**Methodology:** Wenjing Chen, Haiyan Lu, Guozhong Zhang.

**Resources:** Guozhong Zhang, Shushen Zheng, Wenjing Shen.

**Supervision:** Wenjing Shen.

**Writing – original draft:** Liyuan Yang, Genqi Lu.

**Writing – review & editing:** Liyuan Yang, Genqi Lu, Jiabao ren and Wenjing Shen.
